# Extensive impact of saturated fatty acids on metabolic and cardiovascular profile in rats with diet-induced obesity: a canonical analysis

**DOI:** 10.1186/1475-2840-12-65

**Published:** 2013-04-15

**Authors:** Silvio A Oliveira Junior, Carlos R Padovani, Sergio A Rodrigues, Nilza R Silva, Paula F Martinez, Dijon HS Campos, Marina P Okoshi, Katashi Okoshi, Maeli Dal-Pai, Antonio C Cicogna

**Affiliations:** 1 School of Physical Therapy, Federal University of Mato Grosso do Sul, Campo Grande, MS, Brazil; 2 Botucatu Medical School, São Paulo State University, Botucatu, SP, Brazil; 3 Botucatu Biosciences Institute, São Paulo State University, Botucatu, SP, Brazil

**Keywords:** Hypercaloric diet, Lipids, Cardiac remodeling

## Abstract

**Background:**

Although hypercaloric interventions are associated with nutritional, endocrine, metabolic, and cardiovascular disorders in obesity experiments, a rational distinction between the effects of excess adiposity and the individual roles of dietary macronutrients in relation to these disturbances has not previously been studied. This investigation analyzed the correlation between ingested macronutrients (including sucrose and saturated and unsaturated fatty acids) plus body adiposity and metabolic, hormonal, and cardiovascular effects in rats with diet-induced obesity.

**Methods:**

Normotensive *Wistar*-*Kyoto* rats were submitted to Control (CD; 3.2 Kcal/g) and Hypercaloric (HD; 4.6 Kcal/g) diets for 20 weeks followed by nutritional evaluation involving body weight and adiposity measurement. Metabolic and hormonal parameters included glycemia, insulin, insulin resistance, and leptin. Cardiovascular analysis included systolic blood pressure profile, echocardiography, morphometric study of myocardial morphology, and myosin heavy chain (MHC) protein expression. Canonical correlation analysis was used to evaluate the relationships between dietary macronutrients plus adiposity and metabolic, hormonal, and cardiovascular parameters.

**Results:**

Although final group body weights did not differ, HD presented higher adiposity than CD. Diet induced hyperglycemia while insulin and leptin levels remained unchanged. In a cardiovascular context, systolic blood pressure increased with time only in HD. Additionally, in vivo echocardiography revealed cardiac hypertrophy and improved systolic performance in HD compared to CD; and while cardiomyocyte size was unchanged by diet, nuclear volume and collagen interstitial fraction both increased in HD. Also HD exhibited higher relative β-MHC content and β/α-MHC ratio than their Control counterparts. Importantly, body adiposity was weakly associated with cardiovascular effects, as saturated fatty acid intake was directly associated with most cardiac remodeling measurements while unsaturated lipid consumption was inversely correlated with these effects.

**Conclusion:**

Hypercaloric diet was associated with glycemic metabolism and systolic blood pressure disorders and cardiac remodeling. These effects directly and inversely correlated with saturated and unsaturated lipid consumption, respectively.

## Background

Excess dietary fats and carbohydrates have been related to obesity and associated comorbidities such as insulin resistance, Type 2 diabetes and hyperglycemia, and cardiovascular effects including elevated blood pressure and cardiac remodeling [1]. To reproduce these effects in experimental models, researchers have been using sugar- and fat-enriched dietary interventions which are referred to as hypercaloric diets (HD) [2-10]. Multiple findings have shown that hypercaloric diets are associated with endocrine disturbances, such as insulin and glucose metabolism disorders [2-9] and cardiac remodeling, substantiated by hypertrophy [2,4-8,10], interstitial fibrosis [3,5-8], and altered molecular expression of contractile proteins, including the synthesis of β-myosin heavy-chain (MHC) isoforms [7].

However, although dietary interventions have been associated with several disturbances in rodents, a clear distinction between the specific effects of each dietary macronutrient in relation to metabolic and cardiovascular disturbances has not been clarified in literature. Recent studies have shown that high fat diets, with high levels of unsaturated [11-13] and/or saturated fatty acids [14-18], induce fat accumulation, metabolic disorders, and cardiac remodeling. Similarly, sugar intake, per se, has been associated with insulin resistance, hypertension, and cardiac effects in rats [19]. Compared to other macronutrients, sugar administration has been associated with increased cardiac remodeling, contractile dysfunction, and significant mortality in response to pressure overload [20-22].

On the other hand, given the common occurrence of obesity in response to hypercaloric interventions, it is important to consider the endocrine role of adipose tissue in coordinating metabolic and cardiac disorders [23,24]. Also, as HD’s have been associated with multiple effects in rodents, a clear distinction between adiposity effects and specific responses from individual dietary macronutrients has not been previously studied. The primary purpose of this study is to investigate the association among body adiposity, dietary macronutrients and metabolic, hormonal and cardiovascular effects in rats submitted to hypercaloric diet using canonical analysis. Canonical correlation analysis is a method for identifying linear combinations of two sets of variables that are highly correlated with each other [25].

## Methods

### Animals and experimental design

Male 60-day old normotensive *Wistar*-*Kyoto* rats were randomly submitted to control (CD group; n = 10) or hypercaloric (HD group; n = 10) diets for 20 weeks. The CD group received commercial rat chow (Purina®, Paulínia/SP, Brazil) and HD animals received five palatable hypercaloric diets (HD1, HD2, HD3, HD4, HD5), alternately administered [6]; each chow was offered for seven days. Animals were individually housed under 22°C to 24°C temperature and 50% to 70% humidity conditions. A time-controlled system provided 12-hour light/dark cycles. All animals had ad libitum access to water and chow (50 g/day).

The experimental protocol was established according to the “*Guide for the Care and Use of Laboratory Animals*” published by the US National Institutes of Health (NIH Publication No. 85-23, 1996 revision) and approved by the Ethics Committee for Animal Experimentation, Botucatu Medical School, UNESP, Brazil.

### Diet characterization

Diets were prepared by adding a mixture of industrialized products and supplementary ingredients to triturated rat chow [4-9]. All diets provided sufficient and similar amounts of vitamins, minerals, and essential amino acids. The hypercaloric diets were composed as follows: HD1: *Labina* rat chow (35%), roasted peanuts (18%), casein (12%), corn oil (8%), chocolate (9%), and corn biscuits (18%); HD2: *Labina* rat chow (44%), roasted peanuts (22%), casein (13%), corn oil (6%), and French fried potatoes (15%); HD4: *Labina* rat chow (37%), roasted peanuts (19%), casein (10%), corn oil (7%), instant noodles (18%), and grated cheese (9%); and HD5: *Labina* rat chow (36%), roasted peanuts (18%), casein (10%), corn oil (8%), condensed milk (16%), and wafer biscuit (12%) [9]. HD3 consisted of *Labina* rat chow without supplementary ingredients; the increased caloric value was due to additional sucrose in water (1.2 kcal/mL). Detailed diet compositions, in terms of fatty acid and sugar subtypes, are described in a previous study [6].

Hypercaloric diets presented higher energetic density than the control diet (4.6 kcal/g vs. 3.2 kcal/g). While HD2 and HD4 only had higher lipid content, HD1 and HD5 also presented important carbohydrate content. Although HD3 was similar in energetic density (4.6 kcal/g) to the other hypercaloric diets, its composition was mainly based on surplus sucrose from a water solution. The hypercaloric diets were also isocaloric with ~30% more energy content than the standard diet and corresponded to interventions from other diet-induced obesity studies [4-9].

### Nutritional, metabolic, and endocrine profiles of the animals

Nutritional and metabolic profiles included adiposity, body weight, calorie intake, feed efficiency, serum glucose, glycemic tolerance, and insulin levels as well as insulin resistance index (HOMA) [26]. To ascertain whether diet was associated with alterations in nutritional performance, food consumption and water intake were measured daily. Calorie intake was calculated weekly as average weekly food consumption × dietary energetic density. With respect to dietary macronutrient composition, relative calorie intake integrated total consumption of each component throughout the experimental period: these were total saturated and unsaturated fatty acids, protein, and sucrose, the main sugar subtype common to hypercaloric diets [6].

Feeding efficiency and the ability to transform consumed calories into body weight were determined with the formula: mean body weight gain (g)/total calorie intake. Body weight (BW) was evaluated once a week. In relation to glycemic tolerance, after fasting for 12 to 15 hours, rats were submitted to an oral glucose tolerance test. Blood samples were drawn from the tail at baseline and after gavage administration of glucose (3 g/kg) [5-8]. Blood samples were collected at 0, 30, 60, 120, and 180 minutes. Glucose levels were determined using an ACCUCHEK GO KIT glucose analyzer (Roche Diagnostic Brazil Ltd., Brazil). Glucose tolerance was defined as the area under the curve (AUC) generated by plotting glycemic responses.

At week 20 of the experiment, after 12 to 15 hours fasting, animals were anesthetized with sodium pentobarbital (50 mg/kg) and killed by decapitation. For biochemical and hormonal analyses, trunk blood was instantly collected; serum was then separated by centrifugation at 3000 *g* for 15 minutes at 4°C and then stored at –80°C for subsequent assessment. Serum glucose was determined with enzymatic colorimetric kits (Kovalent Diagnosis, Rio de Janeiro, RJ, Brazil). Spectrophotometry was performed with a Micronal, model B 382 spectrophotometer. Serum insulin and leptin were measured by ELISA using assay kits from Linco Research Inc. (St. Charles, MO, USA). Insulin resistance (as defined by the homeostasis model assessment insulin resistance index; HOMA) was calculated as fasting glucose (mg/dl) × fasting insulin (ng/dl)/22.5 [26].

After thoracotomy and abdominal incision, adipose deposits from retroperitoneal and epididymal sites were measured [6]. Adiposity index (AD) was calculated from the sum of the individual fat pad weights: [epididymal fat (EF) + retroperitoneal fat (RPF)]/(body weight – sum of fat pads) × 100 [14].

### Cardiovascular profile

#### Blood pressure and echocardiography

Systolic blood pressure (SBP) was measured at two moments, before and after the experimental period by noninvasive tail-cuff method using a Narco Biosystems Electro-Sphygmomanometer (International Biomedical, Austin, TX, USA) [27]. Each animal was individually coupled to the system and the average of 2 readings recorded for each measurement.

At follow-up (20 weeks), all animals were weighed and anesthetized with ketamine hydrochloride (50 mg/kg) and xylazine hydrochloride (1 mg/kg), administered intramuscularly. Subsequently, animals were positioned in the left lateral position for echocardiography, which was performed with a commercially available echocardiography machine (Sonos 5500, Philips, Andover, MA, USA) equipped with a 12-MHz phased array transducer. All measurements were obtained by the same observer using the method recommended by the American Society of Echocardiography [28].

#### Morphological study

The heart was removed and dissected at the time of euthanasia. A partial section from the left ventricle (~300 to 400 μg from the posterior wall) was used for histological analysis.

Myocardial samples were fixed with a 10% formol solution for 48h and subsequently embedded into paraffin blocks for morphometric analysis. Histological sections (7 μm) stained with hematoxylin-eosin were used to measure the cross-sectional areas of the cardiomyocytes. Cross-sectional area, determined for at least 100 myocytes per slide, was used as an indicator of cell size [7,29].

Major and minor nuclear diameters were measured to determine nuclear volume [30,31]. Fifty nuclei from each animal were measured. The nuclear volume was estimated from the formula for a prolate ellipsoid: V = πAB^2^/6; where A is the major diameter and B is the minor diameter [30,31].

Interstitial collagen fraction was determined by analyzing picrosirius red stained myocardium sections using polarized light [6,7,29]. Histological images were obtained using a LEICA DM LS microscope at 40X magnification, coupled to a computer equipped with Image Pro-plus, an image analysis program (Media Cybernetics, Silver Spring, Maryland, USA). Cardiac tissue components were identified according to color: red for collagen fibers, yellow for myocytes, and white for interstitial space. The digitized profiles were entered into a computer which calculated collagen volume fraction as the sum of all connective tissue areas divided by the sum of all connective tissue plus cardiomyocyte areas. On average, 35 microscopic fields were analyzed using a 320X lens. Perivascular collagen was excluded from this analysis [29].

#### Myosin heavy chain composition

An additional 200 to 300 mg sample from the anterior left ventricle wall was selected and frozen for myocardial MHC evaluation by electrophoresis. Detailed sample preparation methods and electrophoresis conditions are presented in a previous study [6].

#### Statistical analysis

Results are expressed as descriptive measures of centralization and variability. Blood pressure, as a function of diet and experimental period, was evaluated by two-way ANOVA for repeated measurements in independent groups. When significant differences were found (P < 0.05), the Bonferroni test was performed.

Comparisons related to morphological results were analyzed by the Student’s *t* test for independent samples. Association between clusters of dependent variables from macronutrients with independent variables from the nutritional profile was determined by multivariate analysis of canonical correlation. Measures of association between groups of dependent and independent variables were presented by the first canonical correlation accompanied by the respective coefficients of linear functional composition. A similar procedure was used for associations between dependent variables, including macronutrients and body adiposity, with independent variables for metabolic and cardiovascular profile [32]. For all statistical procedures, the significance level was considered to be 5%.

## Results

Although experimental groups were not nutritionally different in relation to final calorie intake or final body weight, feeding efficiency and adiposity were higher in HD rats than their control counterparts (Table 1). Similarly, groups differed as to macronutrient calorie consumption; HD animals presented higher sucrose and saturated and unsaturated fatty acid intake (p < 0.001) and lower protein consumption than CD animals (p < 0.05). In relation to metabolic and hormonal parameters, although insulin, HOMA index, and leptin were similar between groups, the hypercaloric diet induced hyperglycemia (CD: 90.8 ± 1.4 vs. HD: 97.6 ± 2.4, p = 0.03) and impaired glycemic tolerance (CD: 27198 ± 451 vs. HD: 31867 ± 1463, p = 0.01; Table 1).

**Table 1:** Nutritional, metabolic and hormonal characteristics

**Variables**	**Groups**		**p-value**
	**CD**	**HD**	
Calorie intake (kcal/day)	80.1 ± 2.8	85.2 ± 3.1	0.336
Total calorie intake (kcal)	9135 ± 1220	11527 ± 1274	<0.001 *
Feeding efficiency (kcal/g)	0.15 ± 0.01	0.17 ± 0.01	0.032 *
Protein (kcal)	3914 ± 137	2919 ± 125	0.032 *
Sucrose (kcal)	629 ± 22	1020 ± 37	<0.001 *
SFA (kcal)	104.4 ± 3.7	488.0 ± 21.3	<0.001 *
UFA (kcal)	389 ± 14	1733 ± 74	<0.001 *
Final body weight (g)	559 ± 23	592 ± 21	0.305
Adiposity index (%)	5.53 ± 0.57	7.42 ± 0.68	0.047 *
Serum glucose (mg/dL)	90.79 ± 1.44	97.56 ± 2.37	0.025 *
Glycemia (AUC)	27198 ± 451	31867 ± 1463	0.008 *
Insulin (ng/dL)	2.62 ± 0.52	2.02 ± 0.35	0.353
HOMA index	10.35 ± 1.97	8.83 ± 1.55	0.552
Leptin (ng/dL)	8.0 ± 1.1	11.2 ± 1.4	0.095

Results expressed as mean ± standard error; SFA: proportional saturated fatty acid intake; UFA: proportional unsaturated fatty acid intake; AUC: area under the glycemic responses curve; HOMA index: homeostasis model assessment insulin resistance index; CD: control diet; HD: hypercaloric diet; * significant differences between groups; Student’s *t* test.

**Figure 1:**
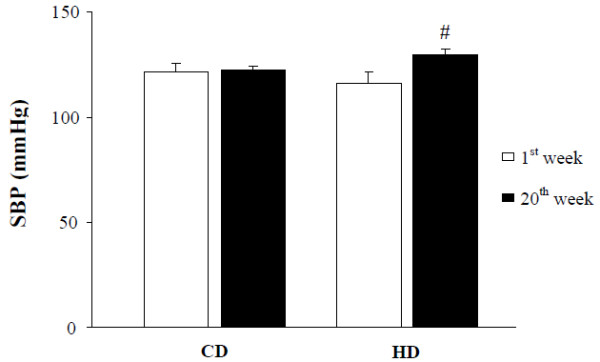
**Systolic blood pressure (SBP) profile as a function of evaluation moment; CD: control diet; HD: hypercaloric diet.** Data are presented as mean ± standard error per group; # p < 0.05 versus 1^st^ week; ANOVA and Bonferroni’s test.

Although arterial pressure was not altered by diet, blood pressure did increase in the HD group over time (CD: 122 ± 14 vs. 123 ± 5 mmHg, p > 0.05; HD: 116 ± 16 vs. 130 ± 8 mmHg, p < 0.05; Figure 1). Echocardiography revealed higher values of left ventricle diastolic thickness and end-diastolic diameter ratio (LVDT/LVEDd) and reduced left ventricular end-systolic diameter (LVESd) in the HD compared to CD group. With respect to functional heart performance, endocardial fractional shortening and ejection fraction were higher in the HD than CD group (Table 2). Histological analysis revealed that, although cardiomyocyte cross-sectional area did not differ between groups, dietary intervention enlarged nuclear volume and interstitial collagen volume fraction in the HD group. Also, β-MHC content was higher in the HD than CD group (Table 3).

**Table 2:** Echocardiography study

**Variables**	**Groups**		**p-value**
	**CD**	**HD**	
Heart rate (beats/min)	280 ± 15	272 ± 11	0.528
LVM (mg)	963 ± 4	955 ± 5	0.814
LVEDd (mm)	8.49 ± 0.15	8.19 ± 0.14	0.070
LVESd (mm)	4.69 ± 0.11	4.12 ± 0.17 *	0.013 *
LVDT (mm)	1.50 ± 0.04	1.57 ± 0.04	0.080
LVDT/LVEDd	0.177 ± 0.005	0.194 ± 0.004 *	0.019 *
LA/AO	1.23 ± 0.07	1.22 ± 0.03	0.425
EFS (%)	44.22 ± 1.31	50.80 ± 2.37 *	0.013 *
Ejection fraction	0.825 ± 0.012	0.870 ± 0.018 *	0.029 *
PWSV (mm/s)	37.4 ± 0.9	37.3 ± 1.0	0.932
E/A	1.74 ± 0.13	1.60 ± 0.09	0.375
EDT (ms)	55.5 ± 3.4	58.5 ± 2.3	0.352
IVRT (ms)	28.3 ± 1.8	30.0 ± 2.0	1.000

Values expressed as mean ± standard error; LVM: left ventricular mass; LVEDd: left ventricular end-diastolic diameter; LVESd: left ventricular end-systolic diameter; LVDT: diastolic thickness of the left ventricle; LVDT/LVEDd: ratio between LVDT and LVEDd; LA: left atrial diameter; LA/AO: ratio between LA and aortic artery diameter; EFS: endocardial fractional shortening; PWSV: posterior wall shortening velocity; E/A: ratio between the E and A waves evaluated in transmitral flow; EDT: E-wave deceleration time; IVRT: isovolumetric relaxation time; * p < 0.05 versus CD; Student’s *t* test.

**Table 3:** Morphological and molecular characteristics of the heart

**Variables**	**Groups**		**p-value**
	**CD**	**HD**	
Nuclear volume (μm^3^)	22.1 ± 2.7	30.4 ± 2.3	0.029 *
CSA (μm^2^)	315 ± 14	319 ± 9	0.811
CIF (%)	3.70 ± 0.38	5.35 ± 0.58	0.028 *
β-MHC (%)	45.4 ± 2.4	55.6 ± 4.1	0.044 *
β/α-MHC ratio	0.87 ± 0.09	1.43 ± 0.21	0.025 *

Results expressed as mean ± standard error; CSA: cross-sectional area of cardiomyocytes; CIF: collagen interstitial fraction; β-MHC: relative left ventricle β- myosin heavy chain isoform content; β/α-MHC ratio: results obtained from relationship between β- and α- MHC expression; CD: control diet; HD: hypercaloric diet; * to significant differences between groups; Student *t*-test.

Canonical analysis revealed a significant correlation between the group of dietary macronutrients and nutritional, metabolic, and hormonal profile variables (r = 0.9712; p < 0.001) (Table 4). Importantly, higher canonical coefficient values revealed a close association between saturated fatty acids and glycemic tolerance. From a cardiovascular aspect, although blood pressure alterations did not show significant correlation with body adiposity or macronutrient subtypes (p > 0.05; Table 5), correlation between macronutrients plus adiposity and in vivo echocardiographic variables was significant (r = 0.9870; p < 0.05). For instance, canonical indexes of association were higher for saturated fatty acid intake and endocardial fraction shortening. Additionally, macronutrients significantly correlated with cardiac histological responses (r = 0.9195; p < 0.005) and myosin heavy chain composition (r = 0.8359; p < 0.05). In both cases, saturated lipid consumption showed a higher canonical coefficient than other macronutrients, while cardiomyocyte nuclear volume (Table 6) and β/α-MHC ratio (Table 7) were the main cardiac effects. Importantly, canonical analysis revealed that unsaturated fatty acid intake was inversely correlated with all these phenotype adaptations (Table 4, 6, 7, 8).

**Table 4:** Measurements of association and canonical correlation between macronutrients and nutritional plus metabolic responses

**Variables**		**Canonical coefficients**
Macronutrients	Protein	−0.0673
	Sucrose	0.4754
	Saturated fatty acids	3.1860
	Unsaturated fatty acids	−3.4308
Nutritional, metabolic and hormonal responses	Feeding efficiency	0.3012
	Body weight	0.0113
	Adiposity	−0.6329
	Serum glucose	0.6065
	Glycemic tolerance	0.2640
	Insulin	0.0091
	HOMA index	−0.0889
Canonical correlation coefficient		0.9712 (p < 0.001)

**Table 5:** Measurements of association and canonical correlation between macronutrients plus adiposity and systolic blood pressure (SBP)

**Variables**		**Canonical coefficients**
Macronutrients and Body Adiposity	Protein	1,2388
	Sucrose	0.8233
	Saturated fatty acids	−19.5616
	Unsaturated fatty acids	20.3829
	Body adiposity	−0.6917
SBP	Final SBP	1.0000
Canonical correlation coefficient		0.4541 (p > 0.05)

**Table 6:** Measurements of association and canonical correlation between macronutrients plus adiposity and echocardiographic indicators

**Variables**		**Canonical coefficients**
Macronutrients and Body Adiposity	Protein	−0.2942
	Sucrose	0.6503
	Saturated fatty acids	19.4472
	Unsaturated fatty acids	−20.1836
	Body adiposity	0.0774
Echocardiographic indicators	Heart rate	0.0253
	LVESd	−0.1195
	Ejection fraction	0.6441
	EFS	0.7212
	Left ventricle mass	−0.0560
	E/A	−0.0002
	LVDT/LVEDd	0.1890
	PWSV	−0.0037
	IVRT	−0.0126
	EDT	0.1017
Canonical correlation coefficient		0.9870 (p < 0.05)

LVESd: left ventricular end-systolic diameter; EFS: endocardium fraction shortening; E/A: ratio between the E and A waves evaluated in transmitral flow; LVDT/LVEDd: ratio between diastolic thickness and end-diastolic diameter of the left ventricle; PWSV: posterior wall shortening velocity; IVRT: isovolumetric relaxation time; EDT: E-wave deceleration time.

**Table 7:** Measurements of association and canonical correlation between macronutrients plus adiposity and myocardial myosin heavy chain composition

**Variables**		**Canonical coefficients**
Macronutrients and Body Adiposity	Protein	−0.8973
	Sucrose	3.8428
	Saturated fatty acids	42.2751
	Unsaturated fatty acids	−46.1834
	Body adiposity	0.1010
MHC variables	β-MHC proportion	−0.3257
	β/α-MHC ratio	0.9454
Canonical correlation coefficient		0.8359 (p < 0.05)

**Table 8:** Measurements of association and canonical correlation coefficients between macronutrients plus adiposity and histological parameters of the heart

**Variables**		**Canonical coefficients**
Macronutrients and Body Adiposity	Protein	−1.3923
	Sucrose	3.3665
	Saturated fatty acids	11.4298
	Unsaturated fatty acids	−15.5675
	Body adiposity	1.0615
Cardiac histology	Cross-sectional area	0.3060
	Nuclear volume	0.9448
	Interstitial collagen	0.1168
Canonical correlation coefficient		0.9195 (p < 0.005)

## Discussion

This is the first paper to document the association between consumption of macronutrients − including protein, sucrose, and saturated and unsaturated fatty acids − and nutritional, metabolic, and cardiovascular responses in rats submitted to a hypercaloric diet. Taking into account the endocrine influence of adiposity on cardiac tissue [23,24], correlation between adiposity and cardiac indicators was also considered. Other major findings were that a hypercaloric diet was related to glycemic disorders, altered blood pressure, and cardiac remodeling, which were more directly associated with saturated fatty acid consumption than sucrose, and inversely correlated with unsaturated lipid intake.

Hypercaloric intervention was obtained by adding a mixture of industrialized products to a standard diet, resulting in enhanced fatty acid content, mainly of unsaturated fats, combined with sucrose overload [4-9]. Although HD’s can induce nutritional, metabolic, and cardiovascular disturbances in rodents, the exact content or fat and sugar composition of these diets have not previously been standardized in literature [1]. While many researchers have employed well-defined, semi-purified diets, in which the fat component replaces carbohydrate and/or protein, other authors have simply added fat and/or sugar to a standard rodent chow; this procedure obviously leads to an unbalanced diet composition with respect to all macronutrients [1]. In our HD model, high fatty acid and carbohydrate content was associated with lower protein intake (Table 2). Rats submitted to a low protein diet presented upregulation of key glucose metabolism components and insulin receptor signal transduction accompanied by cardiac remodeling and heart dysfunction [33]. Thus, reduced protein intake could be associated with diverse effects from HD intervention; however our study did not statistically confirm this. Reductions in dietary protein content are nutritionally important when the protein-to-calorie load ratio is less than the control diet, resulting in attenuated structural development [34]. Importantly, the protein load to energy ratio was similar for both diet groups [6].

Since HD group animals were submitted to a higher dietary offering of fats and sugar, they showed increased intake of fatty acids, both saturated and unsaturated, and sucrose, which were associated with higher feeding efficiency, adiposity, and glycemic disturbance levels (Table 2). Previous studies have revealed that similar interventions increase feeding efficiency, inducing higher adiposity and glycemic disorders in rats after periods of 15 [4,8,9], 16 [2], 20 [5,7], and 32 weeks [10]. However, all these studies present limitations concerning the relative importance of specific macronutrients as causes of nutritional and metabolic disturbances. The results of this study showed that sucrose and especially saturated lipids were strongly correlated with nutritional and metabolic parameters. Dietary saturated fatty acid overload stimulates adipose hypertrophy, due to a rapid process of absorption in adipocytes [35-37]. Consequently, this process induces adipocyte hypertrophy and the production of inflammatory cytokines, which can shift a mechanism towards hyperglycemia and insulin resistance, as well as hypertension, atherosclerosis, and cardiac remodeling [23,24,37].

From a cardiovascular aspect, systolic blood pressure was affected by the passage of time in the HD group, despite final arterial pressure being unchanged. Given that correlation between macronutrients, adiposity, and blood pressure was not significant, other factors, including inflammatory and/or neurohormonal agents may be associated with this hemodynamic response [24]. Hypercaloric high-fat diets have been associated with impaired cardiovascular recovery from stress and the development of arterial hypertension at night, the time when rats are behaviorally active; animals presented unchanged resting blood pressure values [38]. However, increased nitric oxide bioavailability in rats submitted to a high-fat diet could represent an adaptive mechanism which counteracts the reported detrimental impact of an excessively high-energy diet on vascular reactivity [39,40]. This improvement in the endothelial NO pathway could also explain the absence of changes in blood pressure between different dietary groups.

Our paper shows that hypercaloric diet is associated with cardiac remodeling, sustained by improved systolic performance, myocardial hypertrophy, interstitial fibrosis, and concomitant synthesis of β-myosin isoforms. Within this context, regularized canonical correlation shows a major potential role for saturated lipids in provoking cardiac morphological alterations. Moreover, statistical analysis corroborated that unsaturated overload protects cardiac morphology. Saturated fatty acids are the primary metabolic fuel for the heart, and excessive lipid accumulation can stimulate mitochondrial overload and molecular mechanisms in the nucleus causing cell hypertrophy, interstitial remodeling, and myosin isoform transition [15,18]. Furthermore, high concentrations of fatty acids can also activate sarcolemmal Ca^2+^ into cytosol and increase the rate of adenosine triphosphate (ATP) hydrolysis, with a positive inotropic effect on myocardial performance [41]. Similarly, Lima-Leopoldo et al. [4] confirmed the occurrence of myocardial abnormalities in intracellular calcium in rats submitted to hypercaloric diet.

From a functional aspect, systolic performance impacts factors such as heart rate, contractility, preload, and afterload [42]. Diet did not change heart rate; other mechanical influences were not directly evaluated. Even so our data suggest that HD reduced LVEDd (p = 0.07) and increased wall thickness (p = 0.08), which could preserve or even decrease preload. Decreased preload would lead to reduced left ventricular ejection fraction [42], which was not found. Therefore, enhanced systolic function may have resulted from modified afterload and left ventricular hypertrophy. Afterload is a mechanical parameter directly influenced by ventricular pressure and diameter, and inversely related to wall thickness [43]. Chronically increased arterial pressure (Figure 1) is generally associated with increased afterload and, consequently, parietal deformation and cardiac hypertrophy [43,44]. In this context, all the in vivo morphological and morphometric evidence is consistent with left ventricular concentric hypertrophy [44]. Additionally, collagen deposition plus cardiac hypertrophy and MHC changes could be responsible for diastolic dysfunction from a restrictive filling pattern [45]. However, with respect to ventricular performance, diet did not alter diastolic function. Therefore, the described morphological and molecular effects could indicate a primary manifestation of cardiac remodeling due to hypercaloric diet.

A clear distinction between different cardiac remodeling effects driven by different high-fat diets from different lipid sources has not previously been studied [35]. Yet, some studies have shown that saturated, more than unsaturated, fatty acids attenuate cardiac remodeling and increase survival in different heart failure models [20,46,47]. Differentially, western diet – that includes high-fat and high-sugar contents – has been shown to impair cardiac fat acids metabolism and contractile efficiency in rats with inter-renal aortic constriction-induced myocardial hypertrophy [48]. Therefore, our study is important because it reveals a strong association between the fat source and cardiovascular parameters in a landscape of nonexistent heart failure. In this context, the link between dietary saturated fatty acid intake and cardiac remodeling is well documented in experimental studies [14-18]. In particular, saturated fatty acids can stimulate adipose metabolism with the involvement of cytokines and neurohormonal factors and these factors can induce concentric cardiac hypertrophy and alter the stress response [35,49,50]. Therefore, these different effects cannot be discarded as potential agents of cardiac alterations in our experimental model. Moreover, although dietary lipids are the primary fuel for generating ATP and contractile force in the normal heart, they also regulate energy metabolism through peroxisome proliferator-activated receptor-α (PPAR-α) and its coactivators [46]. PPAR-α controls the expression of proteins involved in fatty acid metabolism and is activated by long-chain fatty acids and elevations in plasma lipids [46]. In diabetic rats, the peroxisomal beta-oxidation and the polyunsaturated fatty acids consume are increased in the myocardial tissue [51]. Also, excessive lipid accumulation in non-adipose tissue increases the intracellular pool of long-chain fatty acyl-CoA, thereby providing fatty acid substrates for non-oxidative processes, including triacylglycerol, diacylglycerol, and ceramide synthesis, which can cause cell dysfunction, insulin resistance, apoptotic cell death, and myosin alterations [46,52].

In contrast, unsaturated fatty acids were inversely correlated with cardiac effects. Indeed, unsaturated lipids have been recommended for the prevention of cardiovascular diseases, based on pre-clinical and clinical studies [53,54] and experimental investigations [55]. However, the effects of different types of unsaturated fats on the heart have not been investigated. For example, dietary fish oil supplement is widely used as a source of n-3 polyunsaturated fatty acids, and contains two distinct n-3 fatty acids, eicosapentaenoic acid (EPA) and docosahexaenoic acid (DHA). Previous studies indicated that the primary cardioprotective effect is attributable to DHA rather than EPA [56].

Therefore, our investigation illustrates that the effects due to hypercaloric diet on cardiac phenotype depend on type, rather than amount of macronutrients consumed. Our results also indicate that additional studies are required to better understand the divergent roles of different macronutrient types in regulating cardiac remodeling, both in physiological and pathophysiological settings.

### Limitations

In this study, rats submitted to hypercaloric diet showed increased intake of fatty acids, both saturated and unsaturated, and sucrose. However, saturated fatty acids intake was more associated to metabolic and cardiovascular disorders than the other macronutrients. Taking into account that the animals were subjected to a simultaneous overload of several macronutrients, we could only establish an association between metabolic and cardiovascular disorders and saturated fatty acids intake, but not a specific causal relationship. Therefore, for future studies, analyzing the influence of isolated overload of saturated fatty acids, sucrose, and unsaturated fatty acids on metabolic and cardiovascular variables should be considered.

## Conclusion

Our results confirm that hypercaloric diet induces glycemic disorders and cardiovascular effects, including alterations in arterial pressure and improved systolic performance accomplished by cardiomyocyte nuclear hypertrophy, interstitial fibrosis, and the synthesis of β-myosin isoforms in the myocardium. In contrast to the initial hypothesis, most metabolic and cardiovascular effects are strongly correlated with saturated fatty acid consumption.

## Abbreviations

CD group: Group of animals submitted to control diet; HD group: Group of animals submitted to hypercaloric diet; HD: Hypercaloric diet; BW: Body weight; MHC: Myosin heavy chain; HOMA: Insulin resistance index; AUC: Area under the curve of glycemic responses from the oral glucose tolerance test; AD: Adiposity index; EF: Epididymal fat; RPF: Retroperitoneal fat; SBP: Systolic blood pressure.

## Competing interests

The authors report that there is no potential conflict of interest relevant to this article.

## Authors’ contribution

SAOJ contributed to the study conception and experimental design, the acquisition, analysis and interpretation of data, and first draft of the manuscript. CRP, NRS, and SAR carried out the statistical analysis. PFM contributed to data collection and interpretation, and manuscript preparation. DHS contributed to acquisition of data. MPO and KO made significant contributions to the critical revision and intellectual content of this manuscript. MDP contributed to acquisition and interpretation of microscopic analyses. ACC contributed to study design, data interpretation, manuscript preparation, and fund collection. All authors approved the final manuscript version.
